# Allelic diversity of MSP1 and MSP2 repeat loci correlate with levels of malaria endemicity in Senegal and Nigerian populations

**DOI:** 10.1186/s12936-020-03563-4

**Published:** 2021-01-13

**Authors:** Mary A. Oboh, Tolla Ndiaye, Khadim Diongue, Yaye D. Ndiaye, Mouhamad Sy, Awa B. Deme, Mamadou A. Diallo, Mamadou S. Yade, Sarah K. Volkman, Aida S. Badiane, Alfred Amambua-Ngwa, Daouda Ndiaye

**Affiliations:** 1 Medical Research Council Unit, the Gambia at the London School of Hygiene and Tropical Medicine, Fajara, The Gambia; 2 Laboratory of Parasitology and Mycology, Aristide Le Dantec University Hospital, Cheikh Anta Diop University of Dakar, PO Box 5005, Dakar, Senegal; 3 Department of Immunology and Infectious Diseases, Harvard School of Public Health, Boston, MA USA

**Keywords:** *msp1*, *msp2*, Alleles, Diversity, Nigeria, Senegal, *Plasmodium falciparum*

## Abstract

**Background:**

Characterizing the genetic diversity of malaria parasite populations in different endemic settings (from low to high) could be helpful in determining the effectiveness of malaria interventions. This study compared *Plasmodium falciparum* parasite population diversity from two sites with low (pre-elimination) and high transmission in Senegal and Nigeria, respectively.

**Methods:**

Parasite genomic DNA was extracted from 187 dried blood spot collected from confirmed uncomplicated *P. falciparum* malaria infected patients in Senegal (94) and Nigeria (93). Allelic polymorphism at *merozoite surface protein* 1 (*msp1*) and *merozoite surface protein*- 2 (*msp2*) genes were assessed by nested PCR.

**Results:**

The most frequent *msp1* and *msp2* allelic families are the K1 and IC3D7 allelotypes in both Senegal and Nigeria. Multiplicity of infection (MOI) of greater that 1 and thus complex infections was common in both study sites in Senegal (Thies:1.51/2.53; Kedougou:2.2/2.0 for *msp*1/2) than in Nigeria (Gbagada: 1.39/1.96; Oredo: 1.35/1.75]). The heterozygosity of *msp1* gene was higher in *P. falciparum* isolates from Senegal (Thies: 0.62; Kedougou: 0.53) than isolates from Nigeria (Gbagada: 0.55; Oredo: 0.50). In Senegal, K1 alleles was associated with heavy than with moderate parasite density. Meanwhile, equal proportions of K1 were observed in both heavy and moderate infection types in Nigeria. The IC3D7 subtype allele of the *msp2* family was the most frequent in heavily parasitaemic individuals from both countries than in the moderately infected participants.

**Conclusion:**

The unexpectedly low genetic diversity of infections high endemic Nigerian setting compared to the low endemic settings in Senegal is suggestive of possible epidemic outbreak in Nigeria.

## Background

Malaria caused by *Plasmodium falciparum* continues to be a significant public health havoc in many endemic countries in tropical and sub-tropical parts of the world [1]. Though preventable, malaria accounted for 228 million cases and a mortality of 405,000, in 2018 alone [2]. During same period, the African region contributed a significant proportion of falciparum malaria cases (93%) and mortality (94%) [2]. Though, progress has been observed in malaria control, but in recent time there has been a plateau in advancement.

Malaria in Africa, majorly cause by *P. falciparum* continues to affect almost all age groups at risk of the disease in endemic areas. Senegal and Nigeria are both located in western part of Africa with different levels of interventions, heterogeneity in endemicity and transmission. In Senegal, malaria prevalence is generally low but, the entire population remain at risk and transmission increases gradually from the northern to the Southern part, corresponding to hyperendemicity from the south (annual incidence > 100/1000 inhabitants) to hypoendemicity in the North (annual incidence < 5/1000 inhabitants), respectively [3]. In 2017, malaria accounted for 395 706 cases, 284 deaths and by 33.45% among children under 5 in Senegal [4], despite intensified malaria control over the last 10 years. While malaria endemicity in Nigeria varies between the six geo-political zones of the country, with south west having a mix of meso- and hypo-endemic (1–50%) situation, the south south, south east, north east and north central uniformly hypo-endemic (20–39%), while the north western part being largely meso-endemic (40–49%). Annually, 40% of Nigeria gross domestic product is spent on malaria control [5], yet the country contribute about 25% of annual global incidence rates.

Hence, an effective malaria vaccine that can be readily available to at risk individuals in endemic areas remains imperative. Nevertheless, this initiative is being impeded by the enormous parasite diversity [6–8], which renders the vaccine almost ineffective in some populations. RTS, S, the only malaria vaccine gives a 30% protection even with multiple booster doses. This has mostly been attributed to the genetic diversity of the parasite, which also affects drug efficacy.

In moderate and high transmission areas such as in Africa, the probability of a person being infected with numerous parasite clones at the same time is very high [9–13]. These clones/strains are often a reflection of the transmission intensity or endemicity of an area and as such, impacts the immune system of persons residing in endemic regions. Ultimately, the interplay between parasite clones and the immune selective pressure has a profound impact on the success of any approved vaccine. Therefore, characterization of malaria parasite populations in different endemic settings (from low to high), becomes much more needed to help in the development of a second-generation vaccines and monitor current interventions. The asexual blood stage antigens merozoite surface protein 1 and 2 (MSP1 and MSP2) along with the glutamate-rich protein (GLURP) are highly diverse antigens being exploited for vaccine advancement [14, 15]. However, they have also been used in various studies in evaluating the different circulating clones of *P. falciparum* and/or determining the impact and progress of malaria intervention [16, 17].

The objective of this study was to compare repeat length polymorphism and genetic diversity of *P. falciparum* isolates from Nigeria and Senegal using the *msp1* and *msp2* genes. In addition, the multiplicity of infection and heterozygosity, both of which reflect the transmission intensity as affected by intervention were evaluated.

## Methods

### Ethical consideration

The study from Nigeria was approved by the Institutional Review Board (IRB/16/347), Nigerian Institute of Medical Research, Lagos and Lagos State Health Service Commission. Ethical approval for Senegal was obtained by by the National Ethics Committee for Health Research of Senegal. In addition, written and/or verbal consent where applicable were obtained from all recruited participants.

### Study sites and samples collection

This study was carried out in two West African countries: Nigeria and Senegal.

In Nigeria, isolates were collected in two Local Government Areas of Lagos states namely Kosofe (06° 28′ N 003° 22′ E) and Ikorodu (06° 33′ N 003° 35′ E) from December 2016–March, 2017. Description of study area has been done in an earlier study [18]. Briefly, Lagos state shares a border with the Republic of Benin and is hypo-endemic in most part with a 1.9% prevalence rate in children age 6–59 months [19]. This is in part due to the expansion of insecticide-treated nets (ITNs) coverage, and active indoor residual spraying (IRS) in many of its LGAs [20].

In Senegal, samples were collected in two areas with different endemicity levels, Kedougou located in the south-east and Thiès in western Senegal in 2016. In Kedougou, malaria transmission is seasonal from July to December, with an entomological inoculation rate (EIR) of 20 to 100 infectious bites/person/year and an incidence > 25 malaria cases per 1000 habitants. While in Thies, the malaria situation is hypo-endemic, with an average 0–20 infectious mosquito bite annually and an incidence of 5–15 malaria cases per 1000 habitants. The malaria seasonal transmission in this area coincides with rainy season which generally last up to 4 months (September to December) [3, 21].

In both countries, a purposeful sampling was employed and only febrile patients (94 from Senegal and 93 from Nigeria) visiting health facilities in these areas during malaria transmission season were recruited. Blood samples were collected on filter-paper from patients who met the following inclusion criteria: residence 15-km radius of health facilities, presence of febrile condition (axillary temperature ≥ 37.5 °C) in the previous 48 h, age ranging from 6 months to 75 years and uncomplicated *P*. *falciparum* malaria with parasite density ≥ 1000 asexual forms per microlitre. Patients who presented signs or symptoms of severe malaria as defined by World Health Organization (WHO) [2] and pregnant women were not included.

### “Pre-molecular” sample processing

Care Start^®^ P.f (Access Bio Inc, USA) malaria RDT was used to initially detect *P. falciparum* following the manufacturer’s instruction, and samples found positive were processed for microscopy. Thin and thick blood films were prepared for each patient’s sample following a previously described protocol [20]. Positive Giemsa stained thick smear were counted against a minimum of 500 leucocytes and parasite density(PD) estimated using the 8000 white blood cells (WBCs)/ul following the formula below:

PD = (estimated parasite count × 8000)/number WBCs

PD was further classified into low (> 500 parasite/µl), moderate (500–< 1000 parasite/µl) and high PD (> 1000 parasite/µl) as per the World Health Organization classification system.

### Molecular sample processing: parasite DNA extraction, species and allelic typing of *P. falciparum msp*1 and *msp*2 genes

Parasite genomic DNA was extracted from filter paper using QIAamp DNA Mini kit (QIAGEN, USA) according to the manufacturer’s instructions. Confirmation of *P. falciparum* isolates was done following the Snonou protocol [21] that targets the 18S rRNA of *P. falciparum* isolates. PCR amplification was carried out in a total volume of 25 µl with 1 µl of extracted DNA and 2 µl of nest 1 amplicon for the primary and nested PCR respectively using the Gotaq Green Master mix (Promega) as detailed in an earlier work [16]. Only confirmed *P. falciparum* (corresponding to 205 bp fragment size) were processed for the polymorphic loci genotyping of *P. falciparum msp*1 block 2 (K1, MAD20 and RO33) and *msp*2 central region (IC3D7 and FC27) following a previously described nested PCR protocol [22, 23] (Additional file 1: Table S1). All PCR reactions were carried out in a final volume of 20 μl containing: 1 μl of gDNA, 6 μl GoTaq Green Master Mix (Promega), 1 μl (0.5 μM) of each primer, and 11 μl nuclease free water. In both rounds of reaction, 1 μl of gDNA and PCR amplicon were used respectively as templates for nest 1 and 2 amplifications.

Cycling conditions for both PCR were as follows: initial denaturation at 95 °C for 5 min, followed by 35 cycles of denaturation at 94 °C for 1 min, annealing at 58 °C for 2 min (61 °C for 2 min for the nested reaction) and extension at 72 °C for 2 min; a final extension was carried out at 72 °C for 3 min. Positives (3D7 and Dd2) and negative (DNase free water) controls were systematically incorporated in each PCR run.

The nested PCR product were resolved in 2% agarose gels electrophoresis stained with ethidium bromide and visualized under UV trans-illumination (VersaDoc^®^, Bio-Rad, Hercules, USA). The sizes of PCR fragments were estimated using 100 bp molecular weight ladder (Maker). Presence of more than one genotype was taken as a polyclonal infection, while a single allele was considered as a monoclonal infection. If fragment sizes were within a 20 bp interval, alleles in each family were considered the same [24].

### Statistical analysis

The online Biostatgv was used for statistical analysis. The Chi square test (χ^2^) was used to compare the frequencies of multiclonal isolates between sites and countries. The mean multiplicity of infection (MOI) was calculated as the quotient of the total number of genotypes for each marker and the number of PCR-positive samples. Thus, MOI was calculated by dividing the total number of alleles detected for *msp*1 and *msp*2 genes by the total number of samples [25]. Student’s t test was used to compare MOI between sites.

The expected heterozygosity (He, which is a measure of genetic diversity) was employed to assess population structure of parasites. Heterozygosity was calculated using the following formula He = [n/(n − 1)] [(1 − ΣPi2)], where n = sample size, Pi = allele frequency as described by [26]. A *p* value of ≤ 0.05 was considered suggestive of a statistically significant difference.

## Results

A total of 187 malaria infected participants were recruited for the study with almost equal proportion from the two countries (94 from Senegal and 93 from Nigeria). The mean age was not different from each country and locality within the countries. Although, participants were randomly enrolled into the study, however, more males (108) partook in the study than females (79) (Table 1).

**Table 1:** Characteristics of the study participants in four different endemic sites located in the two West African countries

Variables	Senegal		Nigeria	
	Thies	Kedougou	Kosofe	Ikorodu
Number (%)	47 (50)	47 (50)	29 (31.2)	64 (68.8)
Mean age (± SD)	26.8 (15.2)	20.4 (13.7)	24 (14.4)	27.8 (18.7)
PD (parasite/μl) mean (range) (Min–max)	27,411.7 (900–164,700)	35,348.9 (1350–207,000)	37,673.9 (34–584,000)	24,981 (149–905,600)
Male	41 (87.2)	23 (48.9)	16 (55.2)	28 (43.8)
Female	6 (12.8)	24 (51.1)	13 (44.8)	36 (56.2)

*PD* parasite density

### Genetic polymorphism of *msp1* and *msp2* genes in Senegal and Nigeria

For *msp*1, gene diversity from the two countries were similar. K1 allelic family was predominant in Senegal and Nigeria with frequencies of 44% and 48%, respectively. RO33 allelic family was less represented in the both country with the frequency of 6% in Senegal and 15% in Nigeria. For polygenomic (complex) infections, K1/RO33 combination was the most frequent in both countries but higher in Senegal (17%), and Nigeria (7%). However, the K1/MAD20/RO33 trimorphic allelic infections were least frequent; Senegal (3%) and Nigeria (2%).

**Fig. 1:**
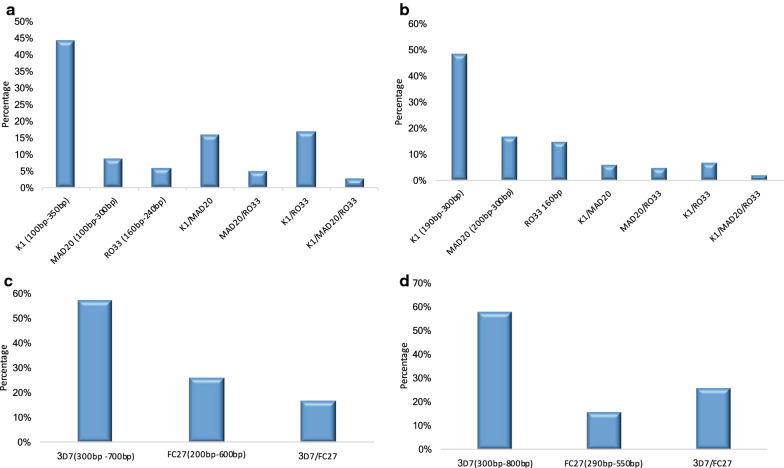
Prevalence of MSP 1 and 2 allele fragment sizes of *P. falciparum; msp 1* in **a** Senegal, **b** Nigeria, and *msp 2* in **c** Senegal, **d** Nigeria

The fragment size of K1 allele type observed in Senegal (100-350 bp) was different from that seen in *P. falciparum* isolates from Nigeria (190-300 bp). Similar pattern was also observed with the MAD20 alleles, where sizes in Senegal ranged from 100-300 bp while those from Nigeria ranged from 200 to 300 bp. In addition, a different pattern was observed in RO33 where only one clone type was seen in Nigeria (160 bp) as against multiple clones in Senegal (160–240 bp) (Fig. 1a, b).

For *msp*2 gene, there was no difference in the frequency of isolates with IC3D7 allele in Senegal (57%) and Nigeria (58%), while the FC27 frequency was more prevalent in Senegal (26%) than in Nigeria (16%). Similar pattern was noticed with the IC3D7/FC27 dimorphic allelic family in both countries. Allele sizes of the IC3D7 variant of *msp*2 ranged from 300 to 700 bp in Senegal and 300–800 bp in Nigerian *P. falciparum* isolates, while FC27 alleles varied in size from 200 to 600 bp in Senegal and 290–550 bp in Nigeria (Fig. 1c, d).

### Multiplicity of *P. falciparum* infection and heterozygosity of *msp*1 and *msp*2

The presence of multiple clones in a single infection define by the MOI index was higher in both study sites in Senegal (Thies, 1.51/2.53; Kedougou, 2.2/2.0 for *msp*1/2) than the sites in Nigeria (Gbagada, 1.39/1.96; Oredo, 1.35/1.75). Consequently, the heterozygosity of *msp*1 gene was higher in *P. falciparum* isolates from Senegal (Thies, 0.62; Kedougou, 0.53) than isolates from Nigeria (Gbagada, 0.55; Oredo, 0.50). However, the heterozygosity of *msp*2 gene was not different for the two countries (Table 2).

**Table 2:** Multiplicity of infection and heterozygosity of *msp*1 and *msp*2 of *P. falciparum* from Senegal and Nigeria

Gene	Senegal		Nigeria		p-value
	Thies	Kedougou	Gbagada	Oredo	
*msp*1					
MOI	1.51	2.2	1.39	1.35	0.00*
He	0.62	0.53	0.55	0.50	0.89
*msp*2					
MOI	2.53	2.00	1.96	1.75	0.39
He	0.48	0.44	0.47	0.48	0.77
MOI (both genes)	2.65	2.68	1.64	1.57	0.72

*MOI* multiplicity of infection, *He* heterozygosity

* p-value less than 0.05 significant

### Genetic polymorphism of *msp*1 and *msp*2 genes by parasite density

The distribution of various allele types in both *msp*1 and 2 in individuals infected with different parasite densities showed some level of stratification. For *msp*1 allele type, K1, MAD20, RO33 and their various allele combinations were not amplified in individuals with low parasitaemia infections (50–499 parasites/µl) from both countries. The K1 allele was more prevalent in individuals with higher parasitaemia infections (28) than those with moderate infections (18) from Senegal. However, in Nigeria, equal proportions of K1 were present in both moderate and high parasitaemic infections. Individuals with heavy infections from Senegal showed the highest proportion of K1/MAD20 (14) allele combination and same pattern was observed for the K1/RO33 mixture (13).

**Fig. 2:**
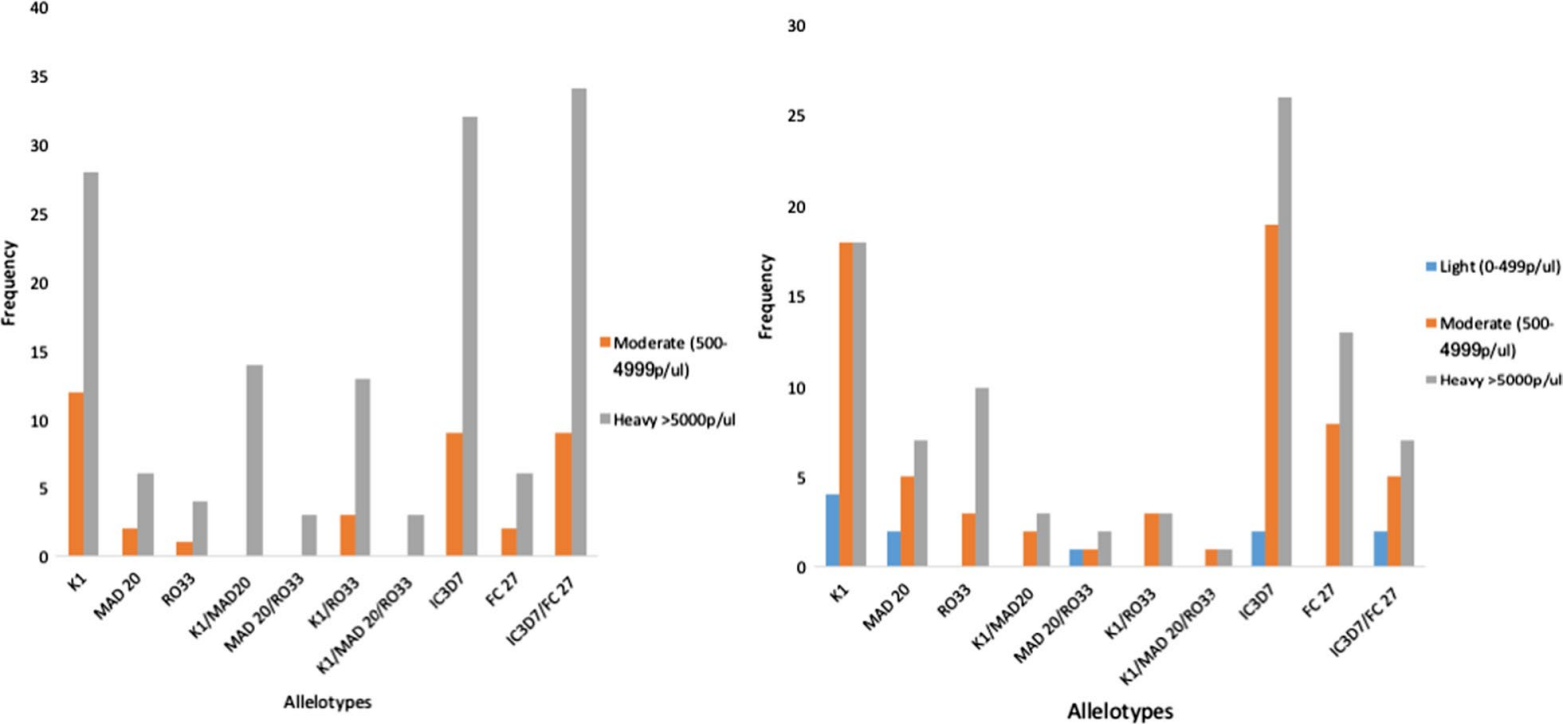
**a** Genetic diversity of *P. falciparum msp*1 and *msp*2 stratified by parasite density in infected individuals from Senegal. **b** Genetic diversity of *P. falciparum msp*1 and *msp*2 stratified by parasite density in infected individuals from Nigeria

The IC3D7 subtype allele of the *msp*2 family showed high occurrence in high parasitaemic individuals from both countries (Senegal, 32; Nigeria, 26) than in the moderately infected participants. Similar pattern was noticed with the FC27 allele (Fig. 2a, b).

## Discussion

Control interventions targeting malaria parasite continues to face multiples hurdles from development of drug resistance in the parasite, insecticide resistance by the vectors, and unavailability of a reliable vaccine to confer the necessary protection against the parasite. Hence, there is need for continuous monitoring and evaluation of the effectiveness of control measures. Here, the diversity of *P. falciparum msp* 1 and *msp* 2 from malaria infected individual’s resident in two West African countries with significantly different levels of overall parasite endemicity; hypo-endemic area in Senegal and a meso-hyper endemic area of Nigeria was evaluated. Multiplicity of infection (MOI) index which is related to the number of clones per infection and usually associated with the level of malaria transmission [27–29] was also calculated. High MOI for both the *msp*1 and 2 genes were observed for both study locations in Senegal, but moderate MOI values were noted in Nigeria. Similar results for Senegal were reported by Niang et al. in 2017 [29]. This observation is unexpected as Senegal is generally categorized as a country under the malaria pre-elimination stage and as such, moderate to low MOI levels were expected. This could have several implications for the malaria control programme in Senegal: firstly, as control managers target a more focal control, parasite could be circulating and transmission going on in other not-targetted areas, subsequently, this high MOI observed and if neglected could lead to extensive parasite recombination and hence further diverse falciparum strains that could pose problems in employing the conventional control methods (use of artemisinin combination therapy). While Nigeria with more intense transmission across all regions should usually show high levels of MOI. However, the detection of low-moderate level of MOI in Nigeria shows that though transmission is high, but same clones of parasites are circulating the area. Hence, similar control strategy can be planned and implemented in the areas. Although, caution is needed when interpreting such results as samples were only collected from western Nigeria. Transmission of malaria in Kedougou is highest in Senegal and this region borders high transmission countries such as Mali and Guinea. On the other hand, the sites in Nigeria from the southern region are in proximity to the large Lagos metropolis where malaria prevalence is relatively low compared to the rest of the country. Low complexity infections have been reported in low prevalence urban dwellings across Africa.

Based on heterozygosity, which measures the level of genetic diversity at polymorphic loci, *msp1* was more diverse in infections from Senegal than those from Nigeria. However, similar levels of diversity for *msp2* was observed in both countries. This is in line with the differences in MOI probably due to differences in urbanization levels and recombination between genetically different clones. Indeed, there was a difference in the clonal structures of all allelic families (K1, MAD20, 3D7 and FC27) from both countries with Senegal showing the more sub-structured *msp1* and *msp2* populations. Nevertheless, similar trends has been observed in the Kingdom of Eswatini where a high genetic diversity was obtained in a low transmission area [30]. Circulation of different fragment sizes of K1 alleles in both countries is indicative of the presence of distinct falciparum clones occurring in both countries. Taken together, this result underscores the need for a more comprehensive evaluation of transmission using different epidemiological tools.

The high occurrence of *msp1* K1 allele in high and low parasite infections from both countries has been previously observed by various studies [31], with this alleles associated more with these levels of parasite density. Whether the high parasite density is driving the selection of K1 or vice versa is yet to be determined. The strong presence of this allelic families was reported by many studies carried out in West Africa [28, 32], western Uganda [33] and Iran [34].

Similarly, high frequency of IC3D7 allele type of *msp2* gene was also observed with both grades of parasite density, while no particular pattern was noticed with the FC27 allelotype. This finding is in agreement with the study of Hamid et al. in 2013 [35] where IC3D7 alleles was more predominant in moderate and heavily infected individuals.

## Conclusion

Taken together, it can be concluded that evidence driving selection of these observed allelotypes in moderate and heavy *P. falciparum* individuals should be evaluated in a bidirectional manner and a more holistic approach should be employed in determining the true epidemiological situation of any malaria endemic country as this will help in a more targeted control measure.

## Supplementary Information

**Additional file 1: Table S1.** Sequences of the primers used to amplify the *msp* 1 and *msp* 2 genes of P. falciparum isolates.

## Data Availability

All data generated or analysed during this study are included in this published article.

## Abbreviations

DNA: Deoxyribonucleic acid
MOI: Multiplicity of infection
MSP: Merozoite surface protein
GLURP: Glutamate rich protein
ITNs: Insecticide-treated nets
IRS: Indoor residual spraying
EIR: Entomological inoculation rate
PCR: Polymerase chain reaction
