# Supplementary material for: Allelic diversity of MSP1 and MSP2 repeat loci correlate with levels of malaria endemicity in Senegal and Nigerian populations
Source: Malar J. 2021 Jan 13;20:38. doi: 10.1186/s12936-020-03563-4 (PMC7805152; doi:10.1186/s12936-020-03563-4)
Supplement: Supplementary file 1 — Additional file 1: Table S1. Sequences of the primers used to amplify the msp 1 and msp 2 genes of P. falciparum isolates. [file 12936_2020_3563_MOESM1_ESM.docx]

Supplemenatry table 1: Sequences of the primers used to amplify the *msp 1* and *msp 2* genes of *P. falciparum* isolates

| Amplification/gene | Primer | Primer sequence |
| --- | --- | --- |
| Primary PCR |  |  |
| msp1 | M1-OR | 5’-CTA GAA GCT TTA GAA GAT GCA GTA TTG-3’ |
|  | M1-OF | 5’-CTT AAA TAG TAT TCT AAT TCA AGT GGA TCA-3’ |
| msp2 | M2R | 5’-ATG AAG GTA ATT AAA ACA TTG TCT ATT ATA-3’ |
|  | M2F | 5’-CTT TGT TAC CAT CGG TAC ATT CTT-3’ |
|  |  |  |
| Secondary PCR |  |  |
| K1 | M1K1R | 5’-AAA TGA AGA AGA AAT TAC TAC AAA AGG TGC-3’ |
|  | M1K1F | 5’-GCT TGC ATC AGC TGG AGG GCT TGC ACC AGA-3’ |
| MAD20 | M1MAD20R | 5’-AAA TGA AGG AAC AAG TGG AAC AGC TGT TAC-3’ |
|  | M1MAD20F | 5’-ATC TGA AGG ATT TGT ACG TCT TGA ATT ACC-3’ |
| RO33 | M1RO33R | 5’-TAA AGG ATG GAG CAA ATA CTC AAG TTG TTG-3’ |
|  | M1RO33F | 5’-CAT CTG AAG GAT TTG CAG CAC CTG GAG ATC-3’ |
| IC3D7 | M2ICR | 5’-AAT ACT AAG AGT GTA GGT GCA TATGCT CCA-3’ |
|  | M2ICF | 5’-TTT TAT TTG GTG CAT TGC CAG AAC TTG AAC-3’ |
| FC27 | M2FCR | 5’-AGA AGT ATG GCA GAA AGT AAC CCT TCT ACT-3’ |
|  | M2FCF | 5’-GAT TGT AAT TCG GGG GAT TCA GTT TGT TCG-3’ |
